# Dordaviprone Maintenance After Allogeneic HCT for High‐Risk Acute Myeloid Leukemia and Myelodysplastic Neoplasm

**DOI:** 10.1002/ajh.70428

**Published:** 2026-06-26

**Authors:** Vijaya Raj Bhatt, Christopher S. Wichman, Alyssa Bouska, Moataz Ellithi, Michael Haddadin, Javeed Iqbal, James E. Talmadge, Lori J. Maness, Krishna Gundabolu

**Affiliations:** ^1^ Division of Hematology‐Oncology, Department of Internal Medicine University of Nebraska Medical Center Omaha Nebraska USA; ^2^ Fred & Pamela Buffett Cancer Center University of Nebraska Medical Center Omaha Nebraska USA; ^3^ Department of Biostatistics University of Nebraska Medical Center Omaha Nebraska USA; ^4^ Department of Pathology, Microbiology, and Immunology University of Nebraska Medical Center Omaha Nebraska USA

**Keywords:** acute myeloid leukemia, allogeneic stem cell transplant, clinical trial, dordaviprone, myelodysplastic syndrome, relapse

## Abstract

Dordaviprone is a first‐in‐class small‐molecule imipridone. In preclinical studies, it is active against leukemia cells harboring a TP53 mutation or complex karyotype and leukemia stem cells while sparing normal bone marrow cells. This study aimed to determine the safety of dordaviprone maintenance after allogeneic hematopoietic cell transplantation (HCT) for high‐risk acute myeloid leukemia (AML) and myelodysplastic neoplasm (MDS). In a Phase I trial (*n* = 20), adults with high‐risk AML or MDS received escalating doses of oral dordaviprone administered weekly for up to 13 four‐weekly cycles (ClinicalTrials.gov ID: NCT03932643). The primary objective was to determine the rate of dose‐limiting toxicities (DLTs) and grade ≥ 3 adverse events (AEs). Participants' characteristics included a median age of 68 years (range: 39–75 years) and high‐risk features such as adverse‐risk AML (50%) or very poor‐risk MDS (75%), TP53 mutations (36%), or measurable residual disease before HCT (41% of AML). A total of 10 patients (one unevaluable) received 250–500 mg weekly doses, whereas the remaining 10 patients received 625 mg weekly doses. Patients received a median of 8.5 cycles (range: 1–13) of dordaviprone. No DLTs or graft failures were noted. Grades 3–4 acute GVHD was noted in 5%. Grade ≥ 3 AEs occurred in 45% (15% related). Grade 3–4 cytopenias were infrequent: anemia (15%), neutropenia (10%), and thrombocytopenia (15%). At 2 years, relapse‐free survival and overall survival were 60% (95% confidence interval [CI]: 42%–86%) and 70% (95% CI: 53%–93%), respectively. Posttransplant dordaviprone maintenance was determined to be safe with low rates of cytopenias and promising efficacy in this high‐risk population.

**Trial Registration:**

ClinicalTrials.gov identifier: NCT03932643

## 1 Introduction

Relapses of high‐risk acute myeloid leukemia (AML) and myelodysplastic neoplasm (MDS) after allogeneic hematopoietic cell transplantation (HCT) represent an area of significant unmet clinical need and a research priority identified by the National Cancer Institute and others [1]. For patients with high‐risk AML and MDS, posttransplant relapses, occurring in up to 40%–60% of patients, remain the principal cause of mortality [2, 3]. Outside of a few exceptions [4, 5], the role of posttransplant chemotherapy maintenance to prevent relapses of leukemia is not well established [6]. Posttransplant maintenance therapy is also associated with a significant risk of myelosuppression and other non‐hematologic adverse events (AEs) [6]. However, chemotherapy agents are frequently utilized off‐label in clinical practices [7], thus signifying an urgent need to develop safe and effective therapeutic strategies.

Dordaviprone (ONC201) is a first‐in‐class small molecule imipridone that acts as a selective antagonist of the G protein‐coupled receptor DRD2 and as an allosteric agonist of mitochondrial protease caseinolytic protease P [8, 9]. In preclinical studies, it has demonstrated activity against leukemia cells, including those with a TP53 mutation or complex karyotype that confers resistance to conventional therapy [10, 11]. It is also toxic to leukemia stem cells while sparing normal bone marrow cells [10]. Several clinical trials in solid and hematologic malignancies have demonstrated that oral dordaviprone has a low toxicity profile and is well tolerated [12, 13, 14]. The profile of this drug is therefore well suited for development to prevent posttransplant relapses of AML and MDS. Here, we report the results of the first trial to use dordaviprone as posttransplant maintenance.

## 2 Methods

### 2.1 Patient Eligibility

Key inclusion criteria included adults aged ≥ 19 years who had undergone HCT within the preceding 6–20 weeks for a history of high‐risk AML or MDS, Karnofsky Performance Scale (KPS) of ≥ 70%, < 5% bone marrow blast, absolute neutrophil count (ANC) greater than 1000/μL, and platelet count ≥ 50 000/μL. High‐risk AML or MDS was defined based on the cytogenetic or molecular features (adverse‐risk AML per 2017 European LeukemiaNet, ELN, risk stratification [15]; poor or very poor risk MDS based on the revised international prognostic scoring system [16]) or based on response (relapsed or refractory to initial treatment or in the case of AML, measurable residual disease, MRD, before or after HCT). Key exclusion criteria included a history of acute graft‐versus‐host disease (GVHD) grades 3/4, use of prednisone at a dose of ≥ 0.25 mg/kg/day, uncontrolled serious infection or major cardiopulmonary conditions, or significant organ dysfunction (e.g., aspartate transaminase, alanine transaminase, or bilirubin greater than two times the upper limit of normal, or creatinine clearance < 30 mL/min).

### 2.2 Study Design and Treatment

This Phase 1 trial enrolled patients at the Fred & Pamela Buffett Cancer Center, University of Nebraska Medical Center. Patients received oral weekly dordaviprone at a starting dose of 250 mg with dose escalation by 125 mg up to a maximum dose of 625 mg weekly, in the absence of any DLTs (3 + 3 design). We used a maximum dose of 625 mg weekly based on the recommended phase 2 dose in solid oncology trials, which is the current dose approved by the Food and Drug Administration (FDA) for glioma [14, 17]. Weekly doses were continued for 13 four‐weekly cycles. Patients underwent blood counts and serum chemistry testing every week for the first cycle, every 2 weeks for the second cycle, then every 4 weeks while receiving the study drug. The protocol encouraged but did not require maintaining the use of immunosuppressants such as a calcineurin inhibitor during the first two cycles of treatment. Patients were monitored for toxicities (using Common Terminology Criteria for Adverse Events, CTCAE version 5.0) through the course of treatment. Data on disease relapse and mortality were captured for 2 years.

The study was registered with the ClinicalTrials.gov identifier (NCT03932643). The study drug was provided by Chimerix Inc., now a part of Jazz Pharmaceuticals. The institutional review board at the University of Nebraska Medical Center approved the study. The study was conducted in accordance with the principles of the Declaration of Helsinki and Good Clinical Practice guidelines. All patients provided written informed consent. All authors reviewed and approved the submitted manuscript. Researchers interested in the deidentified individual participant data should contact the corresponding author or the University of Nebraska Medical Center Investigator‐Initiated Trial Office at iitoffice@unmc.edu within 2 years after the publication date. A Data Use Agreement will be required.

### 2.3 Study Endpoints

The primary endpoints were dose‐limiting toxicities (DLTs) during the first cycle and Grade ≥ 3 AEs. DLT was defined as a Grade ≥ 3 non‐hematologic toxicity, confirmed Hy's law cases of severe drug‐induced liver injury, ANC less than 500/μL lasting > 1 week despite myeloid growth factor support, and platelet count < 10 000/μL lasting > 1 week despite transfusion support. The following AEs were not considered a DLT: Grade 3 fatigue, asthenia, fever, anorexia, or constipation; Grade 3 nausea, vomiting, or diarrhea not requiring tube feeding or total parenteral nutrition or requiring or prolonging hospitalization; infection, bleeding, or other expected direct complications of cytopenias due to active underlying AML or MDS; or Grades 3 or 4 isolated electrolyte abnormalities that lasted < 72 h.

Secondary endpoints included overall AEs, relapse, relapse‐free survival (RFS), non‐relapse mortality (NRM), and overall survival (OS). RFS was defined as the time from HCT to relapse or death. NRM was defined as mortality while in remission. OS was defined as the time from HCT to death from any cause. To capture expanded measures of tolerability, we included exploratory endpoints such as changes in health‐related quality of life (HRQOL) and functional status from enrollment to the end of 1 and 3 months after treatment initiation. HRQOL was captured using the Functional Assessment of Cancer Therapy‐Bone Marrow Transplant (FACT‐BMT). Functional measures included KPS, instrumental activities of daily living (self‐reported physical function), and a short physical performance battery (objective physical function).

### 2.4 Statistical Plan

In this Phase 1 trial, we planned to enroll a total of 20 patients, with an expectation that the first 9–12 patients would receive escalating doses of dordaviprone and the remaining patients would receive the maximum tolerated dose or 625 mg weekly.

The efficacy and safety analyses were done in the full set of 20 patients, all of whom received at least one dose of the study drug. To be considered evaluable for DLT, a patient should have either completed the first cycle of treatment (4 weekly doses) without DLT or encountered DLT before completing the first cycle. We used the Kaplan–Meier method to estimate RFS and OS distributions. We used cumulative incidence methods to estimate the distribution of relapse and NRM, with death without relapse and relapse as competing events, respectively. We summarized all AEs at the patient level using descriptive statistics. Changes in HRQOL from study enrollment to 1 and 3 months were assessed with the Wilcoxon sign‐rank test. We used the following cut‐offs to determine a clinically meaningful change (improvement or worsening) in functional status from baseline to 3 months after treatment: a minimal change of ≥ 10 for KPS [18] and ≥ 1 point for instrumental activities of daily living [19] and short physical performance battery [20].

## 3 Results

### 3.1 Baseline Characteristics

**FIGURE 1:**
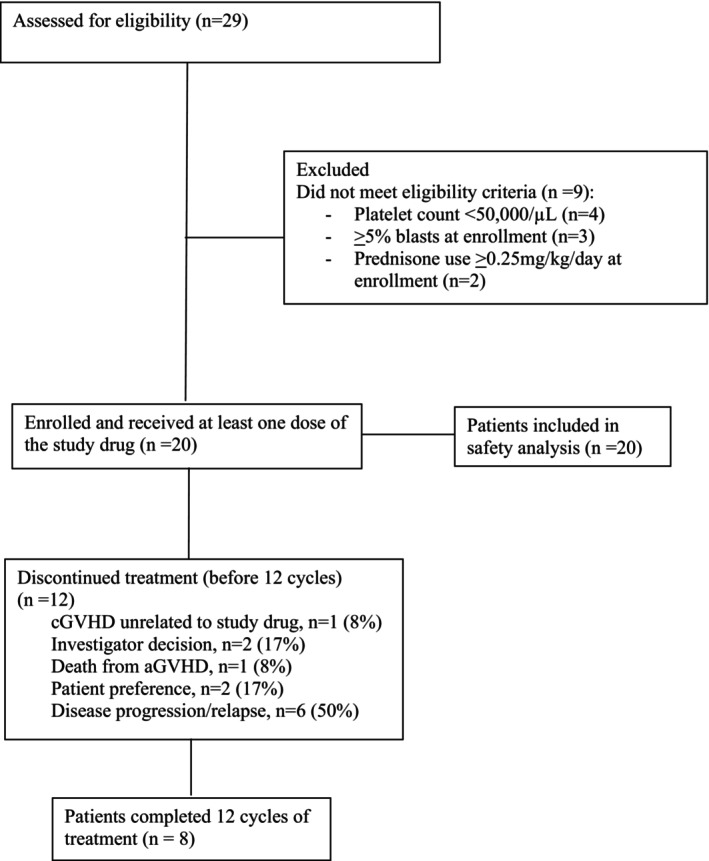
CONSORT diagram showing patients' disposition. aGVHD, acute graft‐versus‐host disease; cGVHD, chronic GVHD; *N*, number of patients.

We consented 29 patients between December 2019 and August 2023; 9 did not meet eligibility criteria because of platelet count < 50 000/μL (*n* = 4), ≥ 5% blasts at enrollment (*n* = 3), and prednisone use at a dose of ≥ 0.25 mg/kg/day (*n* = 2) (Figure 1). Patients were enrolled at a median of 11.5 weeks from transplant (range: 6–20 weeks), with 80% being enrolled within 12 weeks. The study participants (*n* = 20) had a median age of 68 years (range: 39–75 years); 40% were > 70 years, 60% were men, 95% were White, and 5% were Asian (Table 1). High‐risk features included the following: 50% of patients with AML had adverse risk; 75% of those with MDS had very poor‐risk disease (Table S1). Thirty‐six percent had a TP53 mutation, and 35% had a monosomal karyotype. Other common high‐risk mutations included RUNX1 (26%), BCOR (21%), U2AF1 (16%), STAG2 (15%), and ASXL1 (10%) (Figure S1). Forty‐one percent of patients with AML had MRD before HCT. KPS was 80 (range: 70–90). Sixty percent had an HCT comorbidity index of ≥ 3, and 40% had an impairment in instrumental activities of daily living. Seventy percent received reduced intensity, and 30% received myeloablative conditioning. GVHD prophylaxis included tacrolimus and methotrexate in all patients, with low‐dose antithymocyte globulin use in 80% of patients (*n* = 18). All patients received peripheral blood grafts, mostly from matched unrelated (55%) or related donors (35%).

**TABLE 1:** Baseline characteristics of the patients.

Characteristics	Total (*N* = 20)
Age in years, median (range)	68 (39–75)
Male, *n* (%)	12 (60%)
Race	
White	19 (95%)
Asian	1 (5%)
Disease, *n* (%)	
Acute myeloid leukemia	12 (60%)
Myelodysplastic syndrome	8 (40%)
Acute myeloid leukemia (ELN 2017 risk categories)	
Favorable	3 (25%)
Intermediate	3 (25%)
Adverse	6 (50%)
MRD positivity pretransplant (out of 12 patients with AML)	5 (41%)
Myelodysplastic syndrome (IPSS‐R categories)	
Very poor	6 (75%)
Intermediate	2 (25%)
Conditioning intensity, *n* (%)	
Reduced intensity	14 (70%)
Myeloablative	6 (30%)
Donor type, *n* (%)	
Related	8 (40%)
Unrelated	12 (60%)
HLA matching, *n* (%)	
Matched sibling	7 (35%)
Matched unrelated	11 (55%)
Other, mismatched	2 (10%)
Stem cell source, *n* (%)	
Peripheral blood	20 (100%)

Abbreviations: ELN, European LeukemiaNet; HLA, human leukocyte antigens; IPSS‐R, international prognostic scoring system‐revised; *N*, number.

### 3.2 Follow‐Up and Treatment Characteristics

The CONSORT diagram shows patient disposition. The median follow‐up was 26.5 months (ranging 8–31 months). Ten patients received doses of 250–500 mg. One patient was considered unevaluable at 250 mg dose level because of drug cessation before the DLT window without encountering a DLT. This patient, who started the drug after the third HCT, developed asymptomatic Grade 4 neutropenia, which resolved within a week (thus not meeting the DLT criteria), following which the patient decided not to receive further study drug treatment. The remaining 10 patients received 625 mg doses. Patients received a median of 8.5 cycles (range: 1–13) of treatment. The most common reason for treatment discontinuation was disease relapse; none of the patients discontinued drug because of AEs. Forty‐five percent of patients (*n* = 9) missed a median of 2 (range: 1–8) doses. Six patients (30%) had a reduction in drug doses by at least one dose level at a median of 5 cycles (range 3–10 cycles) of treatment; 3 of these 6 patients subsequently increased the drug dose.

### 3.3 Safety

No DLTs were noted. One patient died (after eight cycles of treatment) from late‐onset acute liver GVHD, likely triggered by the tacrolimus taper and not likely attributed to the study drug. Grades 1–2 acute GVHD was noted in 25%, with Grade 4 acute GVHD noted in only one aforementioned patient (5%). Chronic GVHD over 2 years was noted in 35% of patients, moderate to severe based on the NIH consensus criteria in 25%. Chronic GVHD involved mostly skin (five out of seven patients) as well as the mouth (*n* = 2), liver (*n* = 2), eyes (*n* = 2), and oral cavity (*n* = 1). No graft failure was noted. Any grade AEs and Grade ≥ 3 AEs occurred in 95% (70% related) and 45% (15% related) of patients, respectively. Serious AE was reported in 40% (15% related) (Table 2). The rates of Grades 3–4 cytopenias were relatively low for a posttransplant population: anemia (15%), thrombocytopenia (15%), and neutropenia (10%). Other Grade ≥ 3 AEs occurring in > 5% of patients included generalized muscle weakness (10%); supraventricular tachycardia; fall; increased alanine aminotransferase, increased alkaline phosphatase, increased aspartate aminotransferase, hypertriglyceridemia, hypotension, and thromboembolic event (each in 5%).

**TABLE 2:** Treatment‐emergent adverse events.

Adverse event	*N* (% out of 20 patients)	Possibly or probably related *N* (% out of 20 patients)
Any grade	19 (95%)	14 (70%)
Grade ≥ 3 adverse event	9 (45%)	3 (15%)
Serious adverse event^a^	8 (40%)	3 (15%)
All grade hematological adverse event		
Platelet count decreased	10 (50%)	3 (15%)
Anemia	4 (20%)	1 (5%)
Neutrophil count decreased	2 (10%)	1 (5%)
All grade non‐hematological adverse event in ≥ 15%		
Creatinine increased	9 (45%)	2 (10%)
Fatigue	7 (35%)	3 (15%)
Nausea	6 (30%)	4 (20%)
Alanine aminotransferase increased	5 (25%)	1 (5%)
Alkaline phosphatase increased	4 (20%)	1 (5%)
Blood bilirubin increased	5 (25%)	2 (10%)
Limb edema	4 (20%)	0
Hypertriglyceridemia	4 (20%)	0
Cough	4 (20%)	1 (5%)
Diarrhea	3 (15%)	0
Fall	3 (15%)	0
Aspartate aminotransferase increased	3 (15%)	1 (5%)
Hypomagnesemia	3 (15%)	0
Generalized muscle weakness	3 (15%)	1 (5%)
Grade ≥ 3 adverse event ≥ 10%		
Anemia	3 (15%)	1 (5%)
Platelet count decreased	3 (15%)	1 (5%)
Neutrophil count decreased	2 (10%)	1 (5%)
Generalized muscle weakness	2 (10%)	1 (5%)

Abbreviation: *N*, number.

^a^ Eight patients had at least one serious adverse event, Grade ≥ 3, with a total of 13 serious adverse events: generalized muscle weakness (*n* = 2), thromboembolic event (*n* = 2), and one episode each of supraventricular tachycardia, rectal hemorrhage, fall, creatinine increase, neutrophil count decrease, platelet count decrease, hypotension, squamous cell carcinoma, and late‐onset acute graft‐versus‐host disease of the liver. One episode each of generalized muscle weakness, decreased neutrophil count, and decreased platelet count was considered related to the study drug.

### 3.4 Efficacy

**FIGURE 2:**
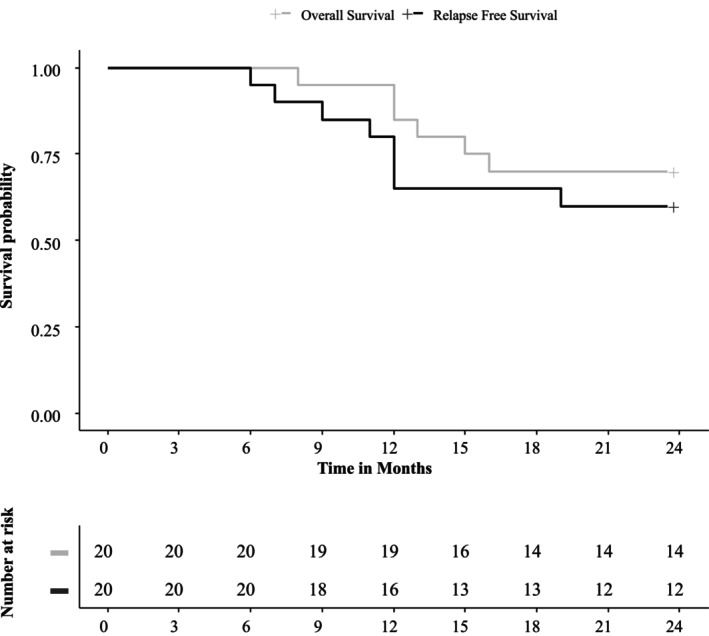
Overall survival (upper curve) and relapse‐free survival (lower curve).

**FIGURE 3:**
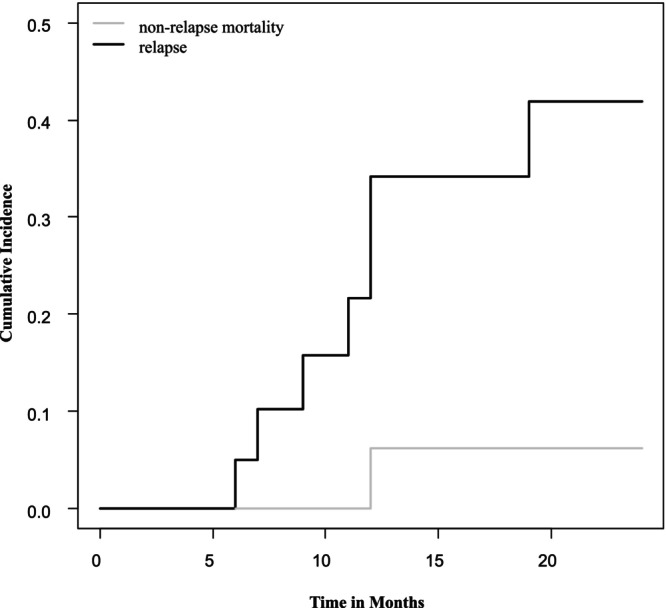
Cumulative incidence of relapse (upper curve) and non‐relapse mortality (lower curve).

At 2 years, RFS and OS were 60% (95% confidence interval [CI]: 42%–86%) and 70% (95% CI: 53%–93%), respectively (Figure 2). The cumulative incidence of NRM was 6.2% (Figure 3). Over the course of 2 years, six patients died mostly from the underlying cancer (*n* = 5), but one patient died of late‐onset acute GVHD of the liver after tacrolimus taper. Two patients had MRD‐positive disease at the time of study enrollment. Whereas both of these patients cleared MRD on restaging of the marrow 3 months later, they subsequently had relapses at 9 and 11 months posttransplant.

With seven patients relapsing (two with AML and five with MDS), the cumulative incidence of relapse was 41.9% at 2 years. Among 12 patients with AML, 2 patients relapsed: a patient with a multihit TP53 mutation and monosomal karyotype and a patient with multiple high‐risk mutations with MRD pre‐ and posttransplant. Among eight patients with MDS, five patients had relapse; all had very high‐risk features: a TP53 mutation (*n* = 1) or monosomal karyotype (*n* = 1) or both (*n* = 3). Out of seven patients with TP53‐mutated AML/MDS, five patients had relapse; one patient came off the trial because of the concern for molecular relapse at 8 months posttransplant, and one patient died of late‐onset acute GVHD of the liver about 11 months after transplant. Three of seven patients with TP53 mutated AML/MDS were alive at 2 years.

### 3.5 HRQOL and Functional Outcomes

**FIGURE 4:**
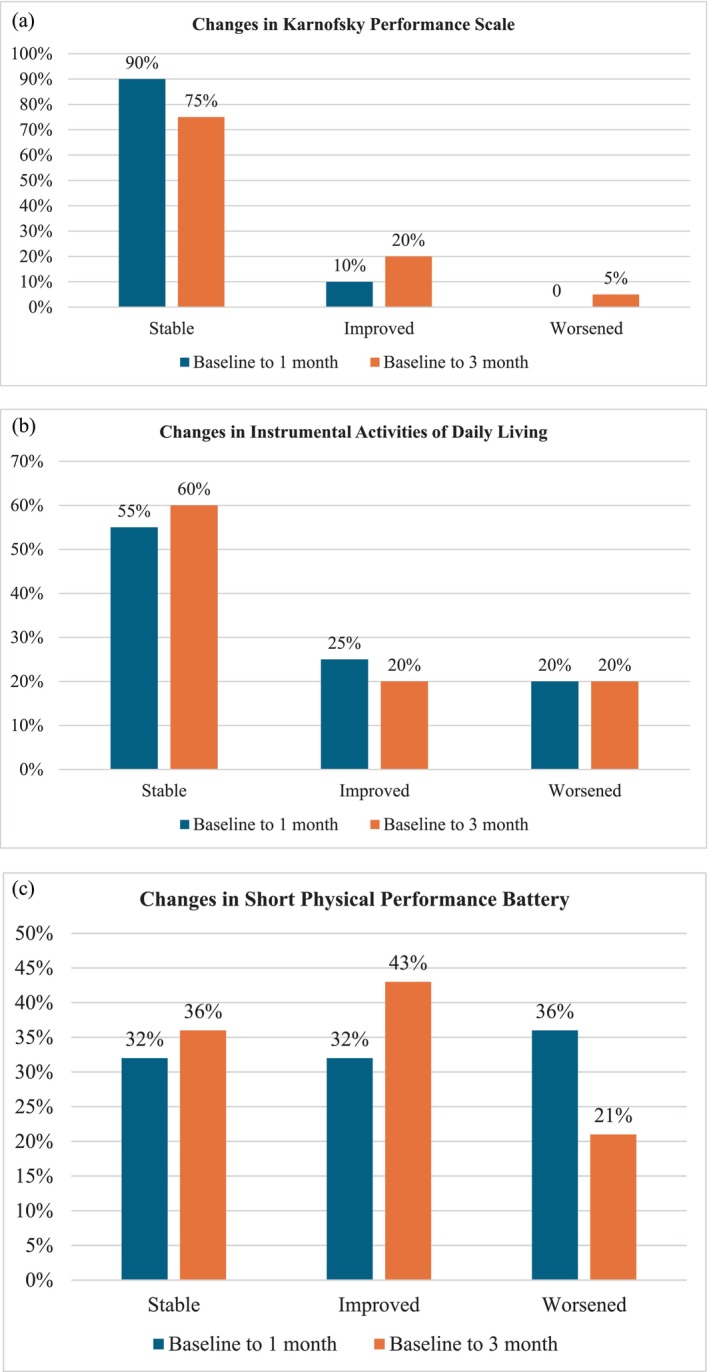
(a–c) Changes in measures of physical function from baseline to 1‐month and 3‐month follow‐up: Physician‐Assessed Karnofsky Performance Scale (a), Self‐Reported Instrumental Activities of Daily Living (b), and Short Physical Performance Battery (c). [Color figure can be viewed at wileyonlinelibrary.com]

The median values of various subscales of FACT‐BMT were similar at baseline, 1 month, and 3 months after treatment initiation (Table S2). The median change from baseline to 1 month and 3 months after treatment initiation was modest, particularly for patients treated with the higher dose of 625 mg (median change by 2–4 points) (Table S3). Most patients had stable or improved measures of physical function from baseline to 3 months, highlighting treatment tolerance. Stabilization or improvement in KPS, instrumental activities of daily living, and a short physical performance battery were noted in 95%, 80%, and 79% of patients, respectively (Figure 4a–c).

## 4 Discussion

The development of novel maintenance strategies to reduce the risk of posttransplant relapses is a key research priority. Although posttransplant maintenance is common in clinical practices [7], randomized data to demonstrate survival benefits is limited to the use of an FLT3 inhibitor or a combination of low‐dose decitabine and recombinant human granulocyte colony‐stimulating factor (GCSF) [4, 5]. In this context, we utilized a first‐in‐class drug in a phase 1 trial, which demonstrated the safety of dordaviprone maintenance therapy after HCT with no DLTs at the highest doses of 625 mg weekly. Dordaviprone was associated with overall low rates of Grades 3–4 cytopenias (10%–15%) and acceptable safety profile (Grade ≥ 3 AEs 45%, 15% related). The NRM was low, particularly for a predominantly older population (median age of 68 years) with many patients having a high comorbidity burden (60% with HCT comorbidity index of ≥ 3) and impaired physical function (40% with impaired instrumental activities of daily living) at enrollment. The safety is also demonstrated by a lack of treatment discontinuation because of AE. The most common reason for treatment discontinuation was disease relapse. The cumulative incidence of relapse was 41.9%. Relapses were more common in patients with MDS, which in part was related to the high prevalence of TP53 mutation and monosomal karyotype. Of 12 patients, 10 with AML did not relapse; many of these patients had MRD pretransplant and one or more high‐risk mutations such as RUNX1, BCOR, STAG2, and other secondary‐type mutations, which confer poor prognosis [21].

Within the limitations of cross‐trial comparisons, the rates of AEs with dordaviprone maintenance were favorable compared to the use of posttransplant azacitidine [6] and comparable to low‐dose decitabine and GCSF [5]. In the MD Anderson trial [6], the use of azacitidine (32 mg/m^2^ per day subcutaneously for 5 days every 28 days, < 50% of the dose used in the pretransplant setting) in 87 patients was associated with 91 Grade ≥ 3 AEs (87% of these were considered related). A total of 58 Grade ≥ 3 AEs were related to bone marrow suppression, which is a major barrier to delivering posttransplant maintenance. In the decitabine GCSF trial (using 5 mg/m^2^ of decitabine on Days 1–5 every 6–8 weeks, 25% of standard dose) [5], Grade ≥ 3 AEs were noted in 38% of patients, and the rate of Grade ≥ 3 cytopenias was 8%–18%. It is also important to note that complications are common in the posttransplant population, as noted in the control arm of the MD Anderson trial (56 Grade ≥ 3 non‐hematological AEs in 94 patients) [6].

The 2‐year OS of 70% was achievable in this high‐risk population with an oral drug administered weekly; hence, the results are considered promising. The demonstration of the safety of an oral drug opens additional options for maintenance therapy alone or in combination with other agents. This is particularly relevant with preclinical evidence of synergy between dordaviprone and hypomethylating agents such as azacitidine [11]. The results of ongoing randomized clinical trials investigating venetoclax in combination with parental azacitidine (NCT04161885) or oral azacitidine (NCT04173533) and potential approval of azacitidine or venetoclax to reduce posttransplant relapses could allow further combination strategies with dordaviprone.

The study has several strengths. This is the first study to report the safety of using a novel first‐in‐class oral drug in the posttransplant maintenance setting. Consistent with the preclinical data, the study demonstrated low cytotoxic effects. The incorporation of HRQOL and functional outcomes provided expanded measures of tolerance, with most patients demonstrating stable or improved HRQOL and functional outcomes following dordaviprone maintenance. The key limitation included a single‐center design and use of in‐house testing for MRD in this investigator‐initiated trial. Even though the cumulative incidence of relapse was high, particularly in patients with MDS, several factors other than maintenance chemotherapy, such as depth of remission at the time of transplant, conditioning intensity, and GVHD prophylaxis, can affect the risk of relapse. A Phase 1 trial is not designed for efficacy analysis; hence, comparison of efficacy outcomes with other randomized Phases 2 or 3 trials may not be appropriate. In addition, the patient characteristics of other trials may be significantly different. For example, only 2% of patients in the decitabine GCSF trial, compared to 36% in this trial, had a TP53 mutation [5]. A future randomized trial will be necessary to determine the efficacy of dordaviprone maintenance to reduce relapse in various subsets of AML and MDS harboring different genetic and mutational changes. The findings of this trial lend to several considerations for future trials. Posttransplant maintenance with dordaviprone may be considered preferentially for high‐risk AML based on the early positive signal in this population, and a separate trial for MDS may be considered subsequently. The randomization should consider factors such as the presence of a TP53 mutation or monosomal karyotype as stratification factors, given poor outcomes of these patients. Alternatively, a separate trial of dordaviprone in combination with another agent should be considered for AML or MDS with a TP53 mutation or monosomal karyotype, where multiple prior strategies have failed to improve outcomes.

In conclusion, the use of dordaviprone posttransplant was well tolerated with no observed DLT and a manageable safety profile. The toxicity profile was not different from complications expected in a posttransplant population. The rates of Grade 3–4 cytopenias were low at 10%–15%. The 2‐year OS of 70% in this high‐risk population is encouraging, particularly in the context of using a weekly oral drug. Given its favorable safety profile, weekly oral dosing, and preclinical synergy with other drugs such as azacitidine [11], this trial demonstrates that dordaviprone is safe for further clinical testing to prevent posttransplant relapses of AML and MDS.

## Author Contributions

V.R.B. conceived the study. V.R.B., C.S.W., L.J.M., and K.G. designed the clinical trial. V.R.B., L.J.M., and K.G. enrolled patients in the clinical trial. C.S.W. performed statistical analysis. V.R.B. wrote an initial draft of the manuscript. All authors contributed to data interpretation, participated in reviewing and editing the manuscript, approved the manuscript for publication, and are accountable for all aspects of the work.

## Funding

This work was supported by the Nebraska Department of Health and Human Services and a Research Support Fund grant from Nebraska Medicine and the University of Nebraska Medical Center.

## Ethics Statement

The study was approved by the institutional review board at the University of Nebraska Medical Center and was conducted according to the Declaration of Helsinki.

## Consent

All study participants signed a written consent to participate in the study.

## Conflicts of Interest

Vijaya Raj Bhatt reports participating in the Safety Monitoring Committee for Protagonist, serving as a member of the National Comprehensive Cancer Network Acute Myeloid Leukemia Panel as a contributor for *BMJ Best Practice*; and receiving consulting fees from Menarini Stemline Therapeutics, Geron, AbbVie, Servier, and Syndax; and research funding (institutional) from Cynata Therapeutics, Pfizer, Jazz, and the National Marrow Donor Program. Michael Haddadin reports participating in the advisory boards for Autolus, Syndax, and Bristol Myers Squibb. Krishna Gundabolu reports participating in the advisory board for Sobi, Syndax, and Incyte. The other authors declare no conflicts of interest.

## Supporting information


**Table S1:** High‐risk features of the participants and associated relapse.
**Table S2:** Changes in Health‐Related Quality of Life, as measured by Functional Assessment of Cancer Therapy‐Bone Marrow Transplantation (FACT‐BMT) scale (actual scores).
**Table S3:** Changes in Health‐Related Quality of Life, as measured by Functional Assessment of Cancer Therapy‐Bone Marrow Transplantation (FACT‐BMT) scale (paired differences from baseline to follow‐up).
**Figure S1:** Mutation profile of the trial participants. Frequency is based on the percentage of positive cases out of those who underwent testing of specific mutations. A total of 19 patients underwent testing for specific mutations. One patient had diagnostic bone marrow at an outside facility, and a mutation panel was not performed. Additional 2–3 patients had myeloid mutation panel that did not include select mutations.

## Data Availability

The data that support the findings of this study are available from the corresponding author upon reasonable request.
