# Supplementary material for: Dordaviprone Maintenance After Allogeneic HCT for High‐Risk Acute Myeloid Leukemia and Myelodysplastic Neoplasm
Source: Am J Hematol. 2026 Jun 26;101(9):2288–96. doi: 10.1002/ajh.70428 (PMC13428374; doi:10.1002/ajh.70428)
Supplement: Supplementary file 1 — Table S1: High‐risk features of the participants and associated relapse. Table S2: Changes in Health‐Related Quality of Life, as measured by Functional Assessment of Cancer Therapy‐Bone Marrow Transplantation (FACT‐BMT) scale (actual scores). Table S3: Changes in Health‐Related Quality of Life, as measured by Functional Assessment of Cancer Therapy‐Bone Marrow Transplantation (FACT‐BMT) scale (paired differences from baseline to follow‐up). Figure S1: Mutation profile of the trial participants. Frequency is based on the percentage of positive cases out of those who underwent testing of specific mutations. A total of 19 patients underwent testing for specific mutations. One patient had diagnostic bone marrow at an outside facility, and a mutation panel was not performed. Additional 2–3 patients had myeloid mutation panel that did not include select mutations. [file AJH-101-2288-s001.docx]

**Supplemental Table 1. High-risk features of the participants and associated relapse**

| **High risk features*** | **Relapse by 2 years** |
| --- | --- |
| **Acute myeloid leukemia (AML)** | |
| Adverse-risk, monosomal karyotype, multiHIT TP53, 13% VAF | **Yes** |
| Adverse-risk, ASXL1, RUNX1, BCOR, STAG2, DNMT3A, IDH2 mutations, MRD pre-transplant and post-transplant | **Yes** |
| Adverse-risk, RUNX1, BCOR, STAG2, U2AF1, DNMT3A, in CR2, MRD positive pre-HCT | No |
| Adverse-risk, RUNX1, U2AF1, NRAS mutations | No |
| Adverse-risk, RUNX1 mutation, IDH2 mutation | No |
| Adverse-risk, RUNX1, BCOR and DNMT3A | No |
| Intermediate-risk, BCOR mutation, MRD pre-transplant | No |
| Intermediate-risk, STAG2, DNMT3A, IDH2 mutations | No |
| Intermediate-risk but CR2, FLT3-ITD, NPM1, DNMT3A, IDH1 | No |
| Favorable-risk (diagnosed with 70,000/ uL WBC), NPM1, IDH1, DNMT3A, NRAS mutations, MRD pre-transplant | No |
| Favorable (biallelic CEBPA mutation with del(7)), WT1, GATA2 mutations, refractory to intensive chemotherapy | No |
| Favorable, tAML with MRD pre-transplant | No |
| **Myelodysplastic neoplasm (MDS)** | |
| Very poor, monosomal karyotype, multiHIT TP53 mutation, 47%VAF, MRD post-transplant | **Yes** |
| Very poor, monosomal karyotype, multiHIT TP53 mutation, 40%VAF | **Yes** |
| Very poor, monosomal karyotype, TP53 mutation with 7.4% VAF (in blood), tMDS | **Yes** |
| Very poor, monosomal karyotype, tMDS**^†^** | **Yes** |
| Intermediate, monosomy 7, TP53 mutation with 8.4% VAF, ASXL1, U2AF1, CEBPA, EZH2, NF1, ETV6, SETBP1 mutations, persistent MDS after HMA treatment | **Yes** |
| Very poor risk, monosomal karyotype, TP53 mutation with 88% VAF | No**^‡^** |
| Very poor, monosomal karyotype, multiHIT TP53 mutation, 40.9% VAF, tMDS | No**§** |
| Intermediate risk but 3rd transplant, post-chemotherapy and transplant failure | No |

CR complete remission, HMA hypomethylating agent, MRD measurable residual disease, tAML treatment-related AML, tMDS treatment-related MDS, VAF variant allele frequency, WBC white blood cell count

*Risk categorization used European LeukemiaNet (ELN) 2017 guidelines for AML, and International Prognostic Scoring System-Revised (IPSS-R) for MDS.

†This is the patient with no available mutation data.

‡Came off the trial because of concern for molecular relapse

§Died about 11 months after transplant

**Supplemental Table 2.** **Changes in Health-Related Quality of Life, as measured by Functional Assessment of Cancer Therapy-Bone Marrow Transplantation (FACT-BMT) scale (actual scores)**

|  | **Screening** | | **1-month follow-up** | | **3-month follow-up** | |
| --- | --- | --- | --- | --- | --- | --- |
| **Scale** | **Higher dose** N = 10 | **Lower dose** N = 10 | **Higher dose** N = 10 | **Lower dose** N = 10 | **Higher dose** N = 8 | **Lower dose** N = 7 |
| Physical well-being | 25.5 (25, 27) | 26 (25, 27) | 25 (25, 27) | 25 (22, 27) | 25 (23, 26.5) | 25 (22, 26) |
| Social well-being | 26 (25, 28) | 26.3 (23, 28) | 25.9 (24, 28) | 23.5 (22, 26) | 24.3 (22.7, 25.8) | 25 (20, 28) |
| Functional Well-Being | 21 (20, 24) | 21.5 (17, 25) | 21 (20, 23) | 19 (16, 22) | 21.5 (17.5, 23) | 21 (20, 23) |
| Emotional well-being | 22.5 (20, 23) | 20.5 (18, 23) | 23 (19, 24) | 20 (18, 22) | 22.5 (20.5, 23) | 19 (17, 23) |
| BMT total score | 29.5 (27, 35) | 29 (24, 32) | 29.5 (28, 31.1) | 28 (22, 30) | 29 (27.5, 31) | 31 (26, 32) |
| FACT-BMT Trial Outcome Index | 78 (70, 83) | 77 (69, 82) | 76 (71, 81) | 74 (60, 80) | 75 (66, 80) | 74 (72, 82) |
| FACT-General total score | 93 (90, 99) | 90 (86, 101) | 92 (89, 94) | 89 (77, 94) | 90 (83, 94) | 92 (84, 95) |
| FACT-BMT total score | 123 (115, 134) | 118 (110, 133) | 122 (116, 124) | 117 (100, 124) | 119 (111, 125) | 121 (115, 125) |

The data shows a summary of median (inter-quartile range) for various FACT BMT subscales.

Higher dose indicates a dose of 625 mg weekly, whereas lower dose refers to 250-500 mg doses.

**Supplemental Table 3.** **Changes in Health-Related Quality of Life, as measured by Functional Assessment of Cancer Therapy-Bone Marrow Transplantation (FACT-BMT) scale (paired differences from baseline to follow-up)**

|  | **1 month-baseline** | | | **3 month-baseline** | | |
| --- | --- | --- | --- | --- | --- | --- |
| **Scale** | **Higher dose N = 10^1^** | **Lower dose N = 10^1^** | **p-value^2^** | **Higher dose N = 8^1^** | **Lower dose N = 7^1^** | **p-value^2^** |
| Physical Well-being | -0.5 (-2, 0) | -0.5 (-3, 1) | 0.79 | 0 (-3.5, 1.5) | -2 (-5, 0) | 0.45 |
| Social Well-Being | 0 (-0.7, 0) | -1.7 (-4, 1.2) | 0.4 | -1 (-2.3, 0.7) | -2.3 (-4, 1) | 0.52 |
| Functional Well-Being | 0 (-2, 2) | -2 (-7, 4) | 0.57 | -1 (-5.5, 4) | -5 (-6, 3) | 0.52 |
| Emotional Well-Being | 0.5 (0, 1) | 0 (-1, 1) | 0.51 | 0.5 (0, 2) | 0 (-1, 2) | 0.41 |
| Bone Marrow Transplant | -1.5 (-3, 2) | -1.5 (-4, 3.3) | 0.76 | 1 (-1, 3) | -1 (-4, 4) | 0.32 |
| FACT-BMT Trial Outcome Index | -2 (-5, 2) | -4 (-12, 8) | 0.6 | -5 (-7, 5) | -9 (-16, 8) | 0.32 |
| FACT-General Total Score | -1 (-6, 3) | -6 (-13, 3) | 0.45 | -4 (-8, 5) | -9 (-17, 5) | 0.35 |
| FACT-BMT Total Score | -2 (-10, 5) | -7 (-14, 6) | 0.52 | -4 (-8, 8) | -14 (-18, 11) | 0.30 |

^1^Median (Quartile1, Quartile3); ^2^Wilcoxon rank sum test unadjusted p-values; all adjusted p-values would be 1.00.

Higher dose indicates a dose of 625 mg weekly, whereas lower dose refers to 250-500 mg doses.

**Supplemental Figure 1. Mutation profile of the trial participants.** Frequency is based on the percentage of positive cases out of those who underwent testing of specific mutations. A total of 19 patients underwent testing for specific mutations. One patient had diagnostic bone marrow at an outside facility, and a mutation panel was not performed. Additional 2-3 patients had myeloid mutation panel that did not include select mutations.
